# Contradiction between genetic analysis and diuretic loading test in type I Bartter syndrome: a case report

**DOI:** 10.1186/s12882-021-02497-6

**Published:** 2021-08-30

**Authors:** Jumpei Kuroda, Ryoko Harada, Riku Hamada, Yusuke Okuda, Yasuhiro Yoshida, Hiroshi Hataya, Kandai Nozu, Kazumoto Iijima, Masataka Honda, Kenji Ishikura

**Affiliations:** 1 Department of Nephrology and Neonatology, Tokyo Metropolitan Children’s Medical Center, 2-8-29 Musashidai, Fuchu, Tokyo, 183-8561 Japan; 2 Department of Nephrology, Tokyo Metropolitan Children’s Medical Center, 2-8-29 Musashidai, Fuchu, Tokyo, 183-8561 Japan; 3 Department of Pediatrics, Kitasato University School of Medicine, 1-15-1 Kitazato, Minami Ward, Sagamihara, Kanagawa 252-0374 Japan; 4 Department of Pediatrics, Graduate School of Medicine, The University of Tokyo, 7-3-1, Hongo, Bunkyo-ku, Tokyo, 113-0033 Japan; 5 Department of Nephrology and General Pediatrics, Tokyo Metropolitan Children’s Medical Center, 2-8-29 Musashidai, Fuchu, Tokyo, 183-8561 Japan; 6 Department of Pediatrics, Kobe University Graduate School of Medicine, 7-5-1 Kusunoki-cho, Chuo-ku, Kobe, Hyogo 650-0017 Japan

**Keywords:** Diuretic test, Type I Bartter syndrome, Atypical cases, Genetic analysis, Case report

## Abstract

**Background:**

In typical cases of Bartter syndrome (BS), assessing response to diuretics (furosemide and thiazide), hereinafter referred to as diuretic loading test, may be used to diagnose the type by detecting which part of the kidney tubule is not functioning correctly. However, the diuretic loading test may not always agree with the results of genetic analyses.

**Case presentation:**

A 5-year-old boy was admitted due to lower extremity weakness and abnormal gait. He had a recurrent episode of muscle weakness and laboratory results showed severe hypokalemia. The direct genomic sequencing of the case revealed a new mutation in the *SLC12A1* gene, which is associated with type I Bartter syndrome. Because there was the difference between the phenotype and genotype, we conducted a diuretic loading test to confirm the diagnosis. However, the results showed a clear increase in urine excretion of Na and Cl. These results were not consistent with typical type I BS, but consistent with the patient’s phenotype.

**Conclusion:**

The diuretic loading test has limited utility for diagnosis especially in atypical cases. On the other hand, this test, which allows assessment of channel function, is useful for better understanding of the genotype-phenotype correlation.

## Background

Bartter syndrome (BS) and Gitelman syndrome (GS) are closely related diseases involving congenital kidney tubular dysfunction characterized by volume depletion, hypokalaemia, and metabolic alkalosis. Owing to recent advances in genetic analysis, the classic classification of BS types, based on clinical symptoms and course, has been superseded by a classification of I to V types, depending on the responsible genes [1–5]. However, a certain latitude is observed in phenotypes even within the same genotype and no one-to-one correspondence has been found between type and clinical manifestation [6, 7]. In typical cases of BS, assessing response to diuretics (furosemide and thiazide), hereinafter referred to as diuretic loading test, may be used to diagnose the type by detecting which part of the kidney tubule is not functioning correctly. However, such tests may not always correspond to the results of genetic analyses. We describe a case of BS diagnosed as type I through genetic analysis that produced a diuretic loading test result apparently inconsistent with this diagnosis.

## Case presentation

A 5-year-old Japanese boy was referred to our hospital by a family doctor. The day before hospitalization, he had pain in both knees, lower extremity weakness, and difficulty walking. Over the previous 6 months, he had had episodes of dorsal foot pain that recurred about once a month and spontaneously abated 1 to 2 days after onset; however, this time, his symptoms had not improved. Blood tests showed pottasium levels of 1.8 mEq/L and creatine kinase levels of 4598 U/L. He was admitted with suspected a recurrent episode of muscle weakness.

The patient had been born without polyhydramnios at a gestational age of 39 weeks and 0 days. His birth length was 52 cm and his birth weight was 3150 g. He screened negative for congenital metabolic abnormalities and had a normal growth and development history. However, he had been prone to polydipsia-polyuria syndrome since birth. His mother had a history of hyperthyroidism and a paternal uncle had a history of nephrotic syndrome.

His vital signs on arrival were normal. He had a normal blood pressure 110/69 mmHg. His extremities showed no obvious muscle weakness, but tenderness was observed in the superior parts of both knee joints. No pain, limitation of motion, swelling, or heat sensation was observed in any other joints. He felt pain when force was applied to his soles as he lay in the supine position. Deep tendon reflexes (patellar and Achilles) were normal.

Blood tests upon admission showed hypokalaemia (K 1.8 mEq/L) and metabolic alkalosis (Venous blood gas; pH 7.528, PCO_2_ 32.7 mmHg, HCO_3_^−^ 27.1 mmol/L). Kidney function was normal (Serum creatinine 0.38 mg/dL). Thyroid function was normal. Plasma renin activity levels were high of 19 ng/mL/hr. (normal value of this age: 1.76 ± 0.99 ng/mL/hr), but aldosterone levels were slightly low of 4.65 ng/dL (normal value of this age: 11.43 ± 6.51 ng/dL), probably because he had treatment of an infusion load. Urine analysis showed hypercalciuria, with a Ca/Cr ratio of 0.49. TTKG and FEK were also high, at 9.5 and 18.9%, respectively. Abdominal ultrasonography revealed hyperechoic areas in the medullae of both kidneys, which was thought to be calcification. There were no abnormal findings in intraperitoneal organs other than the kidneys.

After admission, KCL was administered 5 mEq/kg/day as oral and intravenous infusion therapy for hypokalaemia. About 3 days after admission, he showed an improvement in potassium level associated with the disappearance of lower extremity pain. Blood pressure was consistently normal. Serum potassium levels were normalized on oral therapy of K 2 mEq/kg/day and spironolactone 1.5 mg/kg/day.

As he had hypokalaemia, hypercalciuria, and metabolic alkalosis caused by kidney excretion but showed normal blood pressure and Mg level (2.8 mg/dL), BS was suspected. Because of his age at onset and the mildness of his symptoms, he was first suspected to have type III BS, but a new mutation (c.2762G > T p.Gly921Val, c.3233C > A p.Thr1078Lys) in the *SLC12A1* gene, which is associated with type I BS, was detected by targeted sequencing and confirmed by Sanger sequencing. Furthermore, The PolyPhen-2 and SIFT programs both predicted that this mutation would cause disease. Based on these results, we diagnosed type I BS associated with a compound heterozygous mutation.

Because the onset and clinical course of this case was different from a typical case of type I BS, the patient underwent a diuretic loading test according to a protocol described elsewhere [5] to confirm the clinical phenotype in terms of tubular function. If this case was a typical case of type I BS, the patient showed no response to furosemide; however, response to furosemide was normal, which was inconsistent with type I BS. The response to thiazide was also normal, which ruled out GS and inconsistent with type III BS (Table 1).

**Table 1:** Results of diuretic loading tests^a^

Furosemide	FECl (%)	FENa (%)	Thiazide	FECl (%)	FENa (%)
Pre-test	0.6	0.3	Pre-test	4.5	3.0
1 h later	8.8	7.1	1 h later	11.6	8.1
2 h later	5.9	4.3	2 h later	9.0	6.2

^a^A typical case of type I BS shows no response to furosemide and a good response to thiazide. Type III BS shows no response to thiazide and a normal but weaker response to furosemide. However, the response to furosemide and thiazide was normal in this case, which was inconsistent with type I BS and type III BS and ruled out GS

## Discussion and conclusions

We had experienced a case of genetically type I BS, but the clinical phenotype was mild, and the diuretic loading test results reflected the clinical phenotype. In some atypical cases of BS, the genotype may appear to diverge from the phenotype. For instance, in this case, the diuretic loading test was unable to confirm the results of genetic analysis with regard to BS type. Type I BS (Online Mendelian Inheritance in Man [OMIM] 601,678) is caused by a mutation in the *SLC12A1* gene that encodes the apical furosemide-sensitive Na–K–2Cl cotransporter (NKCC2). In type I BS, as a result of NKCC2 damage, urine excretion of Na and Cl generally does not increase with the administration of furosemide. However, our results showed a clear increase in these markers. We reasoned that as ours was a mild case, NKCC2 dysfunction might be only partial, resulting in a modest response to furosemide. Up to now, diuretic loading tests have generally been considered useful in diagnosing type I BS. However, in fact, there have been no large-scale studies involving diuretic loading tests in patients genetically diagnosed with type I BS; therefore, the usefulness of diuretic loading tests in mild cases such as ours remains open to question.

Another reason for the discrepancy we observed between genetic and diuretic loading tests might be the lack of standard protocols and interpretation guidelines for diuretic loading tests. In many previous studies, patients preparing for such tests were administered fresh water or a dilute saline solution to ensure sufficient urinary flow [5]. However, in our case, blood potassium levels were low, and we were concerned that the test might trigger severe hypokalaemia, so we changed the protocol to include oral administration of KCl and an infusion of electrolytes prior to the test. Therefore, we cannot rule out the possibility of potassium and chloride affecting the diuretic test results. Furthermore, there are currently no clear standards for the dose of diuretics to be administered or for the definition of a reaction. We therefore need to continue gathering data on how the results of diuretic loading tests are affected by individual patient characteristics.

Our experience shows that in BS, the genotype of BS cannot always be diagnosed from the phenotype. Other recent reports and review have also indicated that the genotype-phenotype correlation is not always exact [8]. Conventionally, type I BS in neonates is regarded as serious, but there are reports of a milder “diuresis-only” phenotype among babies delivered at full term with normal birth weight and normal development [6, 7]. In addition, type III BS was hitherto regarded as a relatively mild form; however, recent data indicate that approximately 30% of cases progress to kidney failure [9]. It thus appears likely that cases of BS, such as ours, in which the genotype does not correlate with the phenotype and diuretic loading are in fact quite common; this makes diagnosis using the conventional five-type classification very problematic. Therefore, in order to make a proper diagnosis of the disease BS, we should consider all variations of the disorder as a single disorder (i.e., salt-losing tubulopathy) and genetic testing should be performed aggressively. To better understand the relationship between genotype, phenotype, and tubular channel function, the results of the diuretic loading test should be collected.

To summarize: in many situations, including mild cases of type I BS such as ours, diuretic loading test results may not correlate with BS genotype; this exposes the limited usefulness of the conventional classification system. The diuretic loading test has limited utility for diagnosis especially in atypical cases. On the other hand, this test, which allows assessment of channel function, is useful for better understanding of the genotype-phenotype correlation. Given the lack of standard protocols and interpretation guidelines for diuretic loading tests, the further data collection of the test could be required.

## Data Availability

The datasets during this case report are available from the corresponding author on reasonable request.

## Abbreviations

BS: Bartter syndrome
FEK: Fractional potassium excretion
GS: Gitelman syndrome
TTKG: Transtubular potassium gradien
